# Recreational off-highway vehicle exposure, safety behaviors and crash experiences among adolescents

**DOI:** 10.1186/s40621-022-00405-6

**Published:** 2022-12-21

**Authors:** Charles A. Jennissen, Sienna E. Schaeffer, Pamela J. Hoogerwerf, Kristel M. Wetjen, Lauren J. Mulford, Katharine L. Champoux, Uche E. Okoro, Gerene M. Denning

**Affiliations:** 1 Department of Emergency Medicine, Roy J. and Lucille A. Carver College of Medicine, University of Iowa, Iowa City, USA; 2 Stead Family Department of Pediatrics, Roy J. and Lucille A. Carver College of Medicine, University of Iowa, Iowa City, USA; 3 Roy J. and Lucille A. Carver College of Medicine, University of Iowa, Iowa City, USA; 4 Injury Prevention and Community Outreach, University of Iowa Stead Family Children’s Hospital, University of Iowa, Iowa City, USA; 5 Division of Pediatric Surgery, Roy J. and Lucille A. Carver College of Medicine, University of Iowa, Iowa City, IA USA

**Keywords:** Adolescents, Crash, Pediatrics, Recreational off-highway vehicles, Seat belts, Side-by-side vehicles, Utility task vehicles

## Abstract

**Background:**

Recreational off-highway vehicles (ROVs), often called utility task vehicles (UTVs), are designed to be driven by those ≥ 16 years and manufacturers recommend passengers be at least 12 years old. This study’s objective was to determine Iowa adolescents’ exposure to ROVs, riders’ use of restraint devices, and crash prevalence.

**Methods:**

Adolescents participating in the Safety Tips for ATV Riders (STARs) program at their schools were anonymously surveyed by the Iowa Off-Road Vehicle Safety Task Force from Fall 2014-Fall 2019. Frequency, bivariate (chi square and Fisher’s exact test) and logistic regression analyses were performed using SAS software, V.9.4.

**Results:**

A total of 4,023 students (9–18 years) from 18 school districts participated. Overall, 68% reported having ridden in an ROV. The proportions having ridden an ROV by where participants lived were farm (85%) > country/not farm (73%) > town (60%), *p* < 0.0001. Of those asked additional ROV questions (*n* = 2152), 39% of ROV riders reported riding at least weekly in the previous 12 months. Of those riding ROVs in the past year, 29% reported having at least one crash. Males and respondents living on farms had higher percentages reporting crashes, as compared to females (31% vs. 24%, *p* = 0.005) and those living elsewhere (35% vs. 24%, *p* = 0.0003). Thirty-seven percent of ROV riders never or almost never wore their seatbelt. Seatbelt use was inversely proportional to age, *p* < 0.001. A higher proportion of females reported always or almost always wearing a seat belt (42% vs. 36%, *p* = 0.0016). Percentages never or almost never wearing seatbelts by residence were farm (47%) > country/not farm (38%) > town (32%), *p* = 0.0005. Almost daily riders and those reporting having been in a crash were both 1.7 times more likely to never or almost never wear a seatbelt as compared to infrequent riders and those without a crash, respectively.

**Conclusions:**

Iowa adolescents frequently ride ROVs and often without a seatbelt, putting them at greater risk for serious injury or death in a crash. Almost 30% of riders reported an ROV-related crash in the past year. Our study identified a high-risk population that could be targeted for ROV safety education and other injury prevention efforts.

## Background

Recreational off-highway vehicles (ROVs) are an emerging safety and public health issue (U. S. Consumer Product Safety Commission 2016; Richardson et al. 2018; Jennissen et al. 2021). Over the past fifteen years, U.S. and worldwide sales of ROVs have continued to rise with purchases reaching 690,000 vehicles in 2020 (Polaris Industries 2021). In fact, the sales of ROVs surpassed that of all-terrain vehicles (ATVs) in 2015 with a widening gap ever since (Polaris Industries 2021). Although the number of ATV-related deaths and injuries remains significant (Jennissen et al. 2021), those due to ROVs are a growing concern especially among children and adolescents (Richardson et al. 2018; Linnaus et al. 2017; Jennissen et al. 2020). In two studies, one in Iowa and one including nine Great Plains/Midwestern states, children < 16 years were 44–49% of ROV-related injury victims, a considerably higher proportion than the percentage of youth among ATV crash victims (24–29%) (Jennissen et al. 2020, 2021; Denning et al. 2014; Denning and Jennissen 2018).

All ROVs are designed for off-road use only and like ATVs, have a propensity for rollovers. ROVs have low pressure tires with deep treads that are constructed to grab off-road terrain but can have unpredictable interactions with roadway surfaces. Unlike ATVs, ROVs have a steering wheel, foot pedals for braking and acceleration, and have bench or bucket seats that allow for the carrying of passengers. Most youth injured in ROVs are passengers. However, a University of Iowa study found 30% of children < 16 years injured in ROV crashes were drivers of the vehicle (Jennissen et al. 2020). Youth were also drivers in over one-fifth of the crashes involving injuries collected by the Consumer Product Safety Commission and comprised 14% of ROV drivers killed on public roads in a national database study (U. S. Consumer Product Safety Commission 2016; Richardson et al. 2018).

ROVs have maximum speeds ≥ 30 mph and most can travel at highway speeds. In many places, ROVs are commonly referred to as utility task vehicles (UTVs), but true UTVs by definition have maximum speeds < 25 mph (Wilson 2015). Because of their high speeds, ROVs are required to have a rollover protective structure (ROPS) with seat belts or harness restraints at each seating position.

Although there are a few youth ROV models available, almost all ROVs purchased and used in the U.S. are for adult operators only. Manufacturers warn consumers with vehicle decals and in the owner’s manuals (Kawasaki Heavy Industries 2012; Polaris Industries 2014, 2021), that their vehicles should not be driven by those < 16 years of age. In fact, manufacturers often recommend that passengers should be at least 12 years old and be able to grip the passenger hand hold while sitting with their back against the seat and both feet flat on the floor (Kawasaki Heavy Industries 2012; Polaris Industries 2014, 2021).

There is a large body of published research regarding youth and ATV exposure, crash risks, and related injury. However, there are very few reports on ROV crash and injury epidemiology and no published data on youth’s ROV exposure, riding behaviors and crash experiences. The objective of this study was to determine Iowa adolescents’ exposure to ROVs, their use of restraint devices, and the crashes they have experienced.

## Methods

### Study population

Iowa students who attended the school-based Safety Tips for ATV Riders (STARs) program (Jennissen et al. 2015) were administered a survey in their classrooms. The program educates students on the ten STARs which are key principles of safe ATV operation. Schools had either directly requested the STARs program or were recruited via communication with district administrators or school nurses. The surveys were completed anonymously with the use of Turning Point®, an audience response system. Survey questions were presented by PowerPoint and photographs of ATVs and ROVs were shown to assure participants were aware of the type of vehicle to which the questions pertained. Iowa students 9–18 years of age from 18 school districts completed the surveys from Fall 2014-Fall 2019. The STARs program was presented, and the survey administered at each school district only once during the study period. No students in the classrooms were specifically excluded from participation and all respondents were unique.

### Survey

A collaborative and iterative process was utilized to develop the survey tool used in the study by members of the Iowa Off-Road Vehicle Task Force. Demographic variables in the survey included sex, age, and where respondents lived (e.g., on a farm). All participants were asked whether they had ever ridden in an ROV and if so, how frequently did they ride. Questions also included how often they had been on an ATV and whether they had been in an ATV crash in the past 12 months. In Spring 2017, additional questions were added to the survey. Specifically, participants were asked (in the past 12 months) the frequency of ROV riding, their use of seat belts, and whether they had been in a crash.

### Data analysis

The anonymous surveys were provided to the primary investigator for analysis. The Institutional Review Board deemed the study exempt. Responses were entered into the web-based survey tool, Qualtrics™, and analyses of the compiled data were performed using SAS, previously “Statistical Analysis System,” version 9.4 (SAS Institute, Cary, NC). Frequencies, chi square and Fisher’s exact tests, and logistic regression analyses were performed. For analysis, the age categories of < 11, 12–13, 14–15 and ≥ 16 years were utilized and for the frequency of ATV riding, “almost daily” and “about once a week” were combined into the “at least weekly” category and “about once a month” and “only a few times” were merged to form the “monthly or less” grouping. For logistic regression analyses of ROV seat belt use, dichotomization was accomplished combining “always” and “almost always” versus the other groups combined and then merging “never” and “almost never” versus the other groups combined. All p values were two-tailed, and a *p* value < 0.05 was considered statistically significant. Missing data were not included in analyses.

## Results

### Demographics and riding frequency/characteristics

A total of 4,023 students in Iowa participated in the survey with 2,152 of these completing the revised version that included additional questions on ROV riding frequency, seat belt use and crashes in the previous 12 months. Respondents were nearly equal by sex and three-quarters were 11–14 years old. See Table 1. Over half (55%) lived in a town, 25% resided in the country but not on a farm, and 20% lived on a farm. Two-thirds (66%) had ridden an ATV in the past 12 months; of these, 40% reported riding at least weekly.

**Table 1:** Demographics and other characteristics of participants who completed the Safety Tips for ATV Riders (STARs) program survey about recreational off-highway vehicles (ROVs)

	Respondents total (*N* = 4023)	Respondents to revised survey (*N* = 2152)
Variable	n (Col%)^a^	n (Col%)^a^
*Sex (n* = *3564)*		
Male	1749 (49)	
Female	1815 (51)	
*Age in years (n* = *3499)*		
< 11	502 (14)	
11	677 (19)	
12	829 (24)	
13	655 (19)	
14	443 (13)	
15	118 (3)	
16	113 (3)	
≥ 16	162 (5)	
*Where they live (n* = *3243)*		
On a farm	648 (20)	
In country/not farm	825 (25)	
In town	1770 (55)	
*How often on an ATV in past 12 months (n* = *3506)*		
Almost daily	496 (14) (21)	
About once a week	435 (12) (19)	
About once a month	435 (12) (19)	
Only a few times	945 (27) (41)	
Not in last 12 months	1195 (34) ––	
*How often on an ROV ever (n* = *3408)*		
Almost daily	436 (13) (19)	
About once a week	356 (10) (15)	
About once a month	482 (14) (21)	
Several times a year or less	1056 (31) (45)	
Never been on an ROV	1078 (32) ––	
*How often on an ROV in past 12 months (n* = *1848)*		
Almost daily		287 (16) (23)
About once a week		198 (11) (16)
About once a month		248 (13) (20)
Several times a year or less		521 (28) (42)
Not in last 12 months		594 (32) ––
*ROV seat belt use in past 12 months (n* = *1190)*		
Always or almost always		429 (36)
More than half the time		137 (12)
Less than half the time		130 (11)
Never or almost never		441 (37)
*ROV crashes in past 12 months (n* = *1189)*		
Yes		340 (29)
Not in last 12 months		81 (7)
Never		768 (65)

*ATV* All-terrain vehicle; *Col%* column percent; *ROV* recreational off-highway vehicle

^a^Column total may not equal group N due to missing data

Over two-thirds (68%) reported having ridden in an ROV at least once in their lifetime. Similarly, 68% of those completing the revised survey stated they had ridden an ROV at least once in the past 12 months, and of these, almost two-fifths (39%) rode an ROV at least weekly. Over one-third (36%) of ROV riders in the previous year always or almost always wore their seat belts or harness system, but a similar proportion (37%) reported never or almost never wearing their restraints. Of those who had been in an ROV in the previous 12 months, 29% reported having been in at least one crash during that time and 36% stated they had been in at least one crash in their lifetime. A crash was defined as having a collision, rolling over or having been ejected from the vehicle.

### Having ever ridden an ROV by demographics and other variables

A higher proportion of males than females (70% vs. 67%) reported having ridden in an ROV (*p* = 0.035). See Table 2. Older children had higher proportions reporting riding ROVs (*p* = 0.0003). Those living in towns (60%) vs. those living in the country but not on a farm (73%) vs. those from farms (85%) had progressively increasing percentages of respondents reporting having ridden ROVs. A striking relationship between ATV use and having ridden an ROV was seen, with 90% of frequent ATV riders (at least weekly in the past year) reporting having ridden an ROV.

**Table 2:** Comparison of students who had ever ridden in a recreational off-highway vehicle (ROV) versus those who had not by demographics and by all-terrain vehicle (ATV) riding as reported on a survey by Safety Tips for ATV Riders (STARs) program participants in Iowa schools

	Ever ridden in an ROV			Logistic Regression	
	Yes	No		Analysis (*N* = 2502)	
Variable	n (Row%)^a^	n (Row%)^a^	*p* Value	aOR^b^	95% CI
*Sex (n* = *3154)*					
Male	1085 (70)	462 (30)	0.035	1.03	0.85–1.24
Female	1071 (67)	536 (33)		1.00 (ref)	
*Age in Years (n* = *3090)*					
≤ 11	653 (64)	371 (36)	0.0003	1.00 (ref)	
12–13	931 (71)	384 (29)		1.40	1.13–1.74
14–15	359 (71)	145 (29)		1.30	0.97–1.73
≥ 16	183 (74)	64 (26)		1.29	0.89–1.89
*Where they live (n* = *2925)*					
On a farm	493 (85)	90 (15)	< 0.0001	1.95	1.45–2.62
In country/not farm	547 (73)	201 (27)		1.59	1.26–2.00
In town	957 (60)	637 (40)		1.00 (ref)	
*Frequency of ATV riding in past 12 months (n* = *3113)*					
At least weekly	766 (90)	83 (10)	< 0.0001	9.92	7.33–13.43
Monthly or less	938 (78)	269 (22)		4.72	3.84–5.79
Not in past 12 months	446 (42)	611 (58)		1.00 (ref)	

*aOR* Adjusted odds ratio; *ATV* all-terrain vehicle; *CI* confidence interval; *ROV* recreational off-highway vehicle; *Row%* row percent

^a^Column total may not equal group N due to missing data

^b^Controlling for all other variables in the table

Logistic regression analysis indicated that participants who lived on farms were nearly twice as likely as those living in town to have ridden an ROV, and those living in the country but not on farms were nearly 1.6 times as likely. Compared to students who had not ridden an ATV in the past year, those riding an ATV at least weekly were about 10 times more likely and those who rode ATVs monthly or less were almost five times as likely to have ever ridden an ROV.

### Frequency of ROV use by demographics and other variables

A higher proportion of male riders reported riding ROVs at least weekly as compared to females (38% vs. 29%, *p* < 0.0001). See Table 3. Students ≥ 16 years also had a higher percentage (43%) that rode ROVs at least weekly as compared those who were younger (*p* = 0.0027). Increasingly higher proportions of participants reported riding ROVs at least weekly when comparing those living in town (21%) with those living in the country but not on a farm (33%) and those living on farms (57%), overall *p* < 0.0001. Similarly, those who had ridden ATVs at least weekly over the past year had higher percentages that rode ROVs at least weekly (60%) as compared to those that did not ride ATVs or did so less frequently (18% and 19%, respectively, *p* < 0.0001).

**Table 3:** Comparison of frequent (at least weekly) versus less frequent (monthly or less) recreational off-highway vehicle (ROV) riders by demographics and by frequency of all-terrain vehicle (ATV) riding as reported on a survey by Safety Tips for ATV Riders (STARs) program participants in Iowa schools

	Frequency of ROV riding			Logistic regression	
	At least weekly	Monthly or less		Analysis (*N* = 1720)	
Variable	n (Row%)^a^	n (Row%)^a^	*p*-Value	aOR^b^	95% CI
*Sex (n* = *2156)*					
Male	413 (38)	672 (62)	< 0.0001	1.31	1.04–1.64
Female	314 (29)	757 (71)		1.00 (ref)	
*Age in years (n* = *2126)*					
≤ 11	228 (35)	425 (65)	0.0027	1.00 (ref)	
12–13	311 (33)	620 (67)		0.95	0.72–1.24
14–15	97 (27)	262 (73)		0.59	0.41–0.85
≥ 16	78 (43)	105 (57)		1.32	0.86–2.01
*Where they live (n* = *1997)*					
On a farm	279 (57)	214 (43)	< 0.0001	2.90	2.18–3.86
In country/not farm	178 (33)	369 (67)		1.37	1.04–1.82
In town	200 (21)	757 (79)		1.00 (ref)	
*Frequency of ATV riding in past 12 months (n* = *2150)*					
At least weekly	462 (60)	304 (40)	< 0.0001	5.31	3.79–7.45
Monthly or less	174 (19)	764 (81)		1.07	0.76–1.50
Not in past 12 months	82 (18)	364 (82)		1.00 (ref)	

*aOR* Adjusted odds ratio; *ATV* all-terrain vehicle; *CI* confidence interval; *ROV* recreational off-highway vehicle; *Row%* row percent

^a^Column total may not equal group N due to missing data

^b^Controlling for all other variables in the table

Males were 1.3 times more likely to be frequent riders (at least weekly) as compared to females. Those living on farms were nearly three times as likely and those living in the country but not on a farm were about 1.4 times as likely to be frequent riders as compared to respondents living in town. Participants reporting frequent ATVs riding (at least weekly) were over five times more likely to report frequent ROV riding as compared to those who had not ridden an ATV in the past year.

### ROV seat belt use by demographics and other variables

A higher proportion of males reported not wearing seat belts when riding in ROVs as compared to females (*p* = 0.0016), with 42% of males stating they never or almost never wore them as compared to 32% of females. See Table 4. Progressively older youth had increasingly greater percentages who reported not wearing seat belts (*p* < 0.0001). For example, 29% of youth ≤ 11 years of age as compared to 65% of students ≥ 16 years indicated they never or almost never wore seat belts when riding in ROVs. Similarly, seat belt use varied by type of residence (overall *p* = 0.0005); never or almost never wearing a seat belt was 47% for students living on a farm, 38% for those living in the country/not on a farm, and 32% for those living in town.

**Table 4:** Comparison of the frequency of seat belt or harness use by demographics, by frequency of recreational off-highway vehicle (ROV) and all-terrain vehicle (ATV) riding, and by whether riders had been in a crash over the indicated time periods as reported on a survey by Safety Tips for ATV Riders (STARs) program participants in Iowa schools

	Frequency of seatbelt/harness use				
	Always or almost always	More than half the time	Less than half the time	Never or almost never	
Variable	n (Row%)^a^	n (Row%)^a^	n (Row%)^a^	n (Row%)^a^	*p* value
*Sex (n* = *1093)*					
Male	197 (36)	56 (10)	65 (12)	235 (42)	0.0016
Female	229 (42)	78 (14)	59 (11)	174 (32)	
*Age in years (n* = *1090)*					
≤ 11	207 (51)	44 (11)	36 (9)	120 (29)	< 0.0001
12–13	149 (34)	64 (15)	64 (15)	163 (37)	
14–15	40 (25)	17 (11)	17 (11)	84 (53)	
≥ 16	16 (19)	6 (7)	8 (9)	55 (65)	
*Where they live (n* = *1022)*					
On a farm	86 (29)	36 (12)	33 (11)	140 (47)	0.0005
In country/not farm	99 (40)	24 (10)	31 (12)	95 (38)	
In town	212 (44)	62 (13)	50 (10)	154 (32)	
*Frequency of ROV riding in past 12 months (n* = *1137)*					
Almost daily	61 (21)	29 (10)	30 (10)	136 (48)	< 0.0001
About once a week	60 (33)	21 (12)	26 (14)	73 (41)	
About once a month	86 (37)	34 (15)	35 (15)	79 (34)	
Several times a year or less	222 (48)	53 (11)	39 (8)	153 (33)	
*Been in an ROV crash in past 12 months (n* = *1149)*					
Yes	83 (26)	39 (12)	40 (12)	163 (50)	< 0.0001
No	354 (43)	98 (12)	88 (11)	284 (34)	
*Frequency of ATV Riding in Past 12 Months (n* = *1096)*					
At least weekly	117 (31)	43 (11)	48 (13)	174 (46)	0.0002
Monthly or less	196 (39)	63 (13)	55 (11)	184 (37)	
Not in past 12 months	108 (50)	26 (12)	22 (10)	60 (28)	
*Been in an ATV crash in past 12 months (n* = *1197)*					
Yes	107 (26)	48 (12)	44 (11)	215 (52)	< 0.0001
No	316 (45)	85 (12)	81 (11)	223 (32)	

*ATV* All-terrain vehicle; *ROV* recreational off-highway vehicle; *Row%* row percent

^a^Column total may not equal group N due to missing data

More frequent ROV riders had higher proportions reporting they never or almost never wore a seat belt, overall *p* < 0.0001. Nearly half of those reporting daily ROV riding said they never/almost never wore them. Those stating they had at least one ROV crash in the past year had lower proportions wearing seat belts as compared to those not reporting a crash, *p* < 0.0001. Half of those who had been in a crash in the past year stated they never or almost never wore a seat belt when riding in an ROV. Additionally, more frequent ATV riders (at least weekly) or ATV riders who reported having been in an ATV crash in the past year had lower percentages of ROV seat belt use as compared to those who had not been on an ATV (*p* = 0.0002) or had not been in an ATV crash (*p* < 0.0001).

Males were 1.4 times more likely than females to report never/almost never wearing a seat belt. See Table 5. With regards to age, 12–13 year olds were 1.5 times as likely, 14–15 year olds were 3.8 times as likely, and ≥ 16 year olds were 4.4 times as likely as those ≤ 11 years old to never/almost never wear their seat belt. Respondents living on farms were 1.5 times more likely than those living in town, the most frequent ROV riders (almost daily) were 1.7 times more likely than infrequent riders, and riders reporting having been in an ROV crash in the past 12 months were 1.7 times more likely than those not in a crash to report never/almost never wearing a seat belt when riding in an ROV.

**Table 5:** Logistic regression analysis of frequency of seat belt or harness restraint use when riding in a recreational off-highway vehicle (ROV) in the past 12 months by demographics, by residence type, by frequency of ROV riding, and by whether the participant had been in an ROV crash as reported on a survey by Safety Tips for ATV Riders (STARs) program participants in Iowa schools

	Logistic regression analysis of seat belt use frequency			
	Never/almost never vs. more than almost never (*N* = 849)		Always/almost always vs. less than almost always (*N* = 849)	
Variable	aOR^a^	95% CI	aOR^a^	95% CI
*Sex (n* = *1093)*				
Male	1.42	1.06–1.92	0.82	0.61–1.11
Female	1.00 (ref)		1.00 (ref)	
*Age in years (n* = *1090)*				
≤ 11	1.00 (ref)		1.00 (ref)	
12–13	1.45	1.04–2.03	0.44	0.32–0.61
14–15	3.79	2.36–6.10	0.30	0.18–0.61
≥ 16	4.44	2.51–7.87	0.23	0.12–0.44
*Where they live (n* = *1022)*				
On a farm	1.46	1.01–2.11	0.70	0.48–1.01
In country/not farm	1.28	0.89–1.86	0.94	0.66–1.34
In town	1.00 (ref)		1.00 (ref)	
*Frequency of ROV riding in past 12 months (n* = *1137)*				
Almost daily	1.68	1.09–2.58	0.54	0.34–0.84
About once a week	1.11	0.70–1.76	0.69	0.47–1.02
About once a month	1.01	0.68–1.52	0.70	0.44–1.11
Several times a year or less	1.00 (ref)		1.00 (ref)	
*Been in an ROV Crash in Past 12 Months (n* = *1149)*				
Yes	1.66	1.18–2.34	0.54	0.38–0.78
No	1.00 (ref)		1.00 (ref)	

*aOR* Adjusted odds ratio; *ATV* all-terrain vehicle; *CI* confidence interval; *ROV* recreational off-highway vehicle; *Row%* row percent

^a^Controlling for all other variables in the table

ROV riders 12 years of age and older were less likely to report always/almost always wearing their seat belt as compared to those ≤ 11 years old, with decreasing likelihood as age increased. The most frequent ROV riders (almost daily) and riders reporting having been in at least one ROV crash in the past 12 months were approximately half as likely to report always/almost always wearing their seat belt as compared to infrequent riders and riders who had not been in an ROV crash, respectively.

### ROV crash in the past year by demographics and other variables

A higher proportion of males indicated they had been in an ROV crash in the past 12 months as compared to females (31% vs. 24%, *p* = 0.005). See Table 6. Students living on farms had higher percentages stating they had been in a crash as compared to those living elsewhere (35% vs. 24%, *p* = 0.0003, data not shown in table). A higher frequency of ROV use was associated with a higher proportion of riders who had experienced an ROV crash in the past year, overall *p* < 0.0001. Nearly three-fifths (56%) of almost daily ROV riders reported having been in a crash as compared to 10% of those who rode several times a year or less. In addition, among participants who reported an ATV crash in the past 12 months or were frequent ATV riders (at least weekly), higher percentages reported having been in an ROV crash in the past year as compared to those not in an ATV crash, or were less frequent or non-riders of ATVs, *p* < 0.0001 in each case.

**Table 6:** Comparison of having been in a recreational off-highway vehicle (ROV) crash in the past 12 months versus not by demographics, by residence type, by frequency of ROV riding and all-terrain vehicle (ATV) riding, and by whether respondents had been in an ATV crash in the last 12 months as reported on a survey by Safety Tips for ATV Riders (STARs) program participants in Iowa schools

	Been in ROV crash in past 12 months			Logistic regression analysis (*N* = 890)	
	Yes	No			
Variable	n (Row%)^a^	n (Row%)^a^	*p* value	aOR^b^	95% CI
*Sex (n* = *1172)*					
Male	180 (31)	402 (79)	0.005	0.88	0.62–1.25
Female	139 (24)	451 (76)		1.00 (ref)	
*Age in years (n* = *1170)*					
≤ 11	118 (26)	328 (74)	0.65	1.00 (ref)	
12–13	129 (28)	335 (72)		1.26	0.87–1.83
14–15	43 (24)	134 (76)		1.04	0.59–1.80
≥ 16	26 (31)	57 (79)		1.30	0.66–2.54
*Where they live (n* = *1090)*					
On a farm	105 (35)	195 (65)	0.0008	0.62	0.41–0.95
In country/not farm	56 (21)	205 (79)		0.60	0.39–0.94
In town	135 (26)	394 (74)		1.00 (ref)	
*Frequency of ROV riding in past 12 months (n* = *1189)*					
Almost daily	156 (56)	123 (44)	< 0.0001	9.92	6.04–16.29
About once a week	71 (38)	115 (62)		5.09	3.03–8.57
About once a month	65 (28)	171 (72)		3.65	2.24–5.94
Several times a year or less	48 (10)	440 (90)		1.00 (ref)	
*Been in an ATV crash in past 12 months (n* = *1197)*					
Yes	220 (49)	231 (51)	< 0.0001	4.44	3.14–6.26
No	83 (11)	663 (89)		1.00 (ref)	
*Frequency of ATV riding in Past 12 months (n* = *1179)*					
At least weekly	167 (42)	233 (58)	< 0.0001	Not used in the analysis	
Monthly or less	116 (22)	407 (78)			
Not in past 12 months	44 (17)	212 (83)			

*aOR* Adjusted odds ratio; *ATV* all-terrain vehicle; *CI* confidence interval; *ROV* recreational off-highway vehicle; *Row%* row percent

^a^Column total may not equal group N due to missing data

^b^Controlling for all other variables in the table except those not used in the analysis as noted

When controlling for other variables, respondents living on farms or in the country but not on a farm were about 40% less likely than those living in town to report having been in an ROV crash. There was a strong association with ROV riding frequency and having had been in an ROV crash. Almost daily riders were nearly 10 times more likely, about once a week riders were five times more likely and about once a month riders were over 3.5 times more likely to report having been in an ROV crash in the past year as compared to riders who rode ROVs several times a year or less. In addition, students who reported having been in at least one ATV crash in the past year were almost 4.5 times more likely to have had at least one ROV crash in the past 12 months as compared to those who had not been in an ATV crash.

## Discussion

Our study found that over two-thirds of adolescents surveyed reported having ridden in an ROV. Of these riders, about 40% reported riding ROVs at least weekly and about 60% stated they rode at least monthly. Unfortunately, students indicated they often rode these vehicles without wearing a restraint system. In fact, only 36% said they always or almost always wore seat belts and nearly 40% stated they never or almost never wore one. These results are particularly concerning given that almost 30% of riders reported they had been in at least one ROV-related crash in the past year.

Males overall rode ROVs more frequently than females and were more likely than females to never or almost never wear a seat belt. Although males rode ROVs more often, the overall percentages of males and females having ever ridden in an ROV were similar (70% vs. 67%). Moreover, when controlling for other variables, there was no difference by sex regarding ever having ridden in an ROV or of having been in an ROV crash in the past 12 months. It should be noted that the proportion of females reported as injured in ROV studies (29–45%) (U.S. Consumer Product Safety Commission 2016; Richardson et al. 2018; Jennissen et al. 2020, 2021; Linnaus et al. 2017) is higher than that commonly seen with ATVs where males typically make up > 85% of victims (Denning and Jennissen 2018, 2016; Denning and Jennissen 2018; Denning et al. 2013a, b). We speculate that this may be due in part to the fact that ROV crashes are more likely than ATV crashes to involve passengers and that females are more likely to be passengers than are males in ROVs (Jennissen et al. 2021).

Adolescents appear to be a particularly vulnerable population with regards to ROVs. As stated earlier, youth are a significant proportion of ROV crash victims accounting for nearly a half of those injured in some reports (Jennissen et al. 2021, 2020). Previous studies have shown that injuries from ROV crashes can be quite severe. A study at the University of Iowa found nearly three-quarters of injured ROV riders were hospitalized, and one-quarter required care in the intensive care unit (ICU) with over one-half (53%) of these being children (Jennissen et al. 2020). Similarly, a pediatric trauma center admitted nearly 80% of their ROV-injured patients with 27% needing ICU care (Linnaus et al. 2017).

Our study found a strong relationship between riding frequency and having had an ROV crash in the past year with daily riders being about 10 times more likely to have experienced a crash than those who rode only several times a year or less. Certainly, the more exposure one has to a risk, the more likely one might experience that outcome. However, these data suggest that a wealth of experience and familiarity with ROVs does not necessarily protect youth from crash and potential injury. Perhaps not surprising, those having had at least one ATV crash in the past 12 months had a significantly higher likelihood of having been in an ROV crash during the same period. We hypothesize that recklessness and a high tendency for risk-taking behavior may play a part in the association of ROV and ATV crashes.

ROVs have a relatively narrow track and high center of mass which makes them vulnerable to rollovers especially on sloped terrain and during sharp turns, even at relatively slower speeds (≤ 20 mph) (Jennissen et al. 2020). Rollovers are the most common causes of ROV-related injuries followed by fall/ejection from the vehicle (U.S. Consumer Product Safety Commission 2016; Jennissen et al. 2020, 2021). Safety experts have been concerned about the lateral stability and vehicle handling characteristics of ROVs (U. S. Consumer Product Safety Commission 2016; U.S Consumer Product Safety Commission 2021). Improvements in ROV safety design and engineering are very much needed and should be strongly encouraged by the industry and enforced by federal regulators.

Adolescents in the study reported a low frequency of seat belt use when riding ROVs, and a significant proportion (37%) never or almost never wore them. In several studies of ROV crash victims, over 90% were not restrained (Jennissen et al. 2020, 2021). Almost three-quarters of those who died in a study of newspaper articles of ROV injuries in nine Midwestern and Great Plains states were ejected from the vehicle (Jennissen et al. 2021). Other studies have found half to three-quarters of those injured or killed in ROV crashes were not wearing a restraint with the vast majority being partially or fully ejected (U.S. Consumer Product Safety Commission 2016; Richardson et al. 2018; Linnaus et al. 2017).

Being unrestrained dramatically increases the risk of ejection and subsequently being struck or pinned by the vehicle (Richardson et al. 2018; Jennissen et al. 2020, 2021). Studies have shown that those pinned by an ROV have higher proportions requiring hospital admission (88% vs. 64%) and mechanical ventilation (19% vs. 2%) as compared those who were not pinned. ROVs weigh up to 1000 pounds. They can transmit significant forces to ejected occupants and are extremely difficult to lift off a pinned victim.

We found that progressively older adolescents had incrementally lower seat belt use in ROVs with over half of those 14 years and older never or almost never wearing their restraints. Of grave concern, those who had been in an ROV crash in the past year were almost 50% less likely to always/almost always wear a seat belt and had a 1.7 times greater likelihood of never/almost never using their restraint. It is critical when riding an ROV to be wearing the seat belt or harness restraint system in order to prevent ejection from the vehicle and to remain within the zone of protection afforded by the ROPS in the event of a rollover. As with operators, no passengers should ride in an ROV if they cannot and are not properly restrained.

## Limitations

This study was conducted in a single Midwest state with minimal ethnic/racial diversity, and our subjects were a convenience sample of youth that participated in the STARs program at their school. Thus, our findings may not be generalizable to other youth populations. We did not question subjects whether they were drivers only, passengers only or both with regards to their ROV riding. Similar to other survey studies, our data may be subject to recall and/or social desirability bias. The fact that the survey was administered anonymously may have reduced the latter.

## Conclusions

Adolescents in our study frequently rode ROVs and often without a seat belt. Lack of seat belt use increases the risk of being ejected from the vehicle during a collision or rollover. Of note, almost 30% of riders reported having been in an ROV-related crash in the past year. These results suggest that youth may engage in unsafe ROV riding behaviors and experience crashes as a result. This further suggests they are a high-risk riding population that may benefit from targeted safety education and other injury prevention efforts.

## Data Availability

Data and materials are available to other parties for research purposes after a data sharing agreement plan is agreed to and signed. Those interested should contact the corresponding author.

## Abbreviations

aOR: Adjusted odds ratios
ATV: All-terrain vehicle
e.g.: Exempli gratia (for example)
ICU: Intensive care unit
ROV: Recreational off-highway vehicle
ROPS: Rollover protective structure
SAS: Previously statistical analysis system
STARs: Safety tips for ATV riders
TM: Trademark
U.S.: United States
